# Effectiveness of Inpatient Psychotherapy for Patients With and Without Migratory Background: Do They Benefit Equally?

**DOI:** 10.3389/fpsyt.2020.00542

**Published:** 2020-06-11

**Authors:** Friederike Kobel, Eva Morawa, Yesim Erim

**Affiliations:** Department of Psychosomatic Medicine and Psychotherapy, University Hospital Erlangen, Friedrich-Alexander University Erlangen-Nürnberg (FAU), Erlangen, Germany

**Keywords:** psychotherapy, effectiveness, psychosomatic treatment, migrants, depression, somatoform disorder, anxiety disorder, posttraumatic stress disorder

## Abstract

**Background:**

Research on the effectiveness of inpatient psychotherapy for migrant patients predominantly concludes that they have greater symptom severity at admission and benefit less from psychotherapy. This study aims to compare symptom severity and effects of psychotherapy regarding depression, somatoform disorders, anxiety disorders, and posttraumatic stress disorder (PTSD) in a sample of patients with and without migratory background (MB).

**Methods:**

Symptom severity of 263 patients (T0, pretreatment) and 256 patients (T1, posttreatment) was assessed using the Patient Health Questionnaire somatization module (PHQ-15), depression module (PHQ-9), and general anxiety disorder module (GAD-7), and the PTSD Checklist (PCL-5). Calculations were made for a completer sample and an intention-to-treat (ITT) sample. To investigate the effectiveness of psychotherapy, we calculated effect sizes (Cohen's d) and clinically significant changes using the reliable change index (RCI).

**Results:**

Patients with MB showed significantly higher symptom burden at admission for somatization (p = 0.025, d = 0.345) and posttraumatic symptoms (p = 0.008, d = 0.424) than patients without MB. At discharge, patients with MB reported significantly higher severity regarding all assessed symptoms (somatization: p = 0.001, d = 0.507; depression: p = 0.045, d = 0.313; anxiety: p = 0.012, d = 0.428; traumatization: p = 0.040, d = 0.329) compared with non-migrant patients. Patients without MB improved significantly regarding all assessed symptoms (somatization: p < 0.001, d = -0.304; depression: p < 0.001, d = -0.692; anxiety: p < 0.001, d = -0.605; posttraumatic symptoms: p < 0.001, d = -0.204). Patients with MB improved significantly concerning depression (p < 0.001, d = -0.649) and anxiety (p = 0.002, d = -0.441). Occurrence of comorbidity was high (87.1% had more than one psychiatric diagnosis). Neurotic, stress-related, and somatoform disorders (F4) and personality disorders (F6) were more frequent among patients with MB.

**Conclusions:**

Patients with MB showed higher symptom severity at admission. Our study demonstrated a significant reduction of depressive and anxiety symptoms among patients with MB receiving psychotherapy. Further research is needed to identify interventions that effectively improve somatization and posttraumatic stress for patients with MB, since these symptoms were not significantly reduced.

## Introduction

The number of migrants is increasing globally, with wars, armed conflicts, lack of economic perspective, and effects of climate change being some of the many triggers for migration movements. In view of a recent increase in migrant collectives in Germany, amounting to 25.5% of the total population, the effectiveness and suitability of mental health services for people with migratory background (MB) is therefore gaining importance (1). The largest migrant groups originate from Turkey (13.3%), Poland (10.8%), the Russian Federation (6.6%), Romania (4.7%), and Italy (4.2%) (1).

Psychosomatic medicine in Germany offers free multimodal and multicomponent treatment for all insured patients and mainly consists of psychotherapy. The tradition of inpatient psychotherapy dates back to the early 1920s, when the first psychoanalytic inpatient therapy ward was established in a Berlin hospital (2). Nowadays, inpatient psychotherapy is widespread and well accepted in Germany and the German-speaking countries, where the vast majority of studies analyzing the effectiveness of inpatient psychotherapeutic treatment have been conducted. Of these studies, some have been carried out in the context of rehabilitation (3), while others have demonstrated the effectiveness of general inpatient psychotherapy (4–9). Liebherz and Rabung (4) conducted a meta-analysis based on a systematic review of 103 studies, showing a medium to large effect of psychotherapeutic inpatient treatment on physical, psychological, cognitive, social, and functional well-being (g = 0.71).

Existing evidence on the association between migration and health is inconsistent. Overall, the majority of studies find that migrant patients show a higher burden of psychological disorders and benefit less from psychotherapy. Mösko et al. (10) and Wiborg et al. (11), for example, indicated higher symptom burdens among migrant patients.

Studies on the outcomes of psychosomatic treatment from rehabilitation settings (10, 12–16), describe migrant patients with higher symptom burdens at admission and benefiting less from psychosomatic treatment than patients without MB. Mösko et al. (10) showed Turkish MB to be an independent negative predictor for treatment success. Likewise, Göbber et al. (12), Brause et al. (13), and Zollmann et al. (16) found that patients with Turkish MB had worse therapy outcomes than patients without Turkish MB. Only one study provides evidence that MB is not negatively associated with therapy outcome (11).

Little research has been done with respect to the prevalence of psychosomatic disorders among migrants. In a representative population-based survey in Germany comparing first-generation migrants and non-migrants, higher risk for depression [odds ratio (OR)=1.24; confidence interval (CI)=1.16–1.17], generalized anxiety (OR=1.38; CI=1.13–1.68), panic attacks in the past four weeks (OR=1.43; CI=1.16–1.17), distressed personality disorder (OR=1.28; CI=1.13–1.45) and suicidal ideation (OR=1.44; CI=1.19–1.74) were detected among first-generation migrants (17). An international study including 23 European countries confirmed that immigrants and ethnic minorities suffered from more depressive symptoms than native study participants (18).

Erim et al. (19) analyzed a sample of Turkish patients in an outpatient psychosomatic setting using Structured Clinical Interview-I (SKID-I) for diagnostics of mental disorders. The most frequent diagnoses in this sample were somatoform disorders (41.2%), depression (37.3%), and PTSD (31.4%). A further study showed differences in the prevalence of diagnoses comparing the two largest migrant groups in Germany, Turkish and Eastern European (20). Patients with Turkish MB suffered significantly more often from mood disorders (18.4%) than patients with an Eastern European background (9.8%). Sariaslan et al. (21) examined patients of Turkish origin in primary care. Depressiveness and somatoform symptoms were significantly higher among this group than among patients of German origin.

Cultural adaption of psychotherapy is known to be very important to therapy outcomes (22–25). Kirmayer et al. (26) implemented a service for cultural consultation in Canada. In Germany, manuals for culturally sensitive approaches have been published by Erim et al. (23) and Machleidt et al. (27). Von Lersner et al. (28) developed guidelines for training in inter-/transcultural competence for psychotherapists. Tantam 2007 (29) referred to “ethnic matching” of patient and therapist as being advantageous for therapy outcome. The treatment provided in this study was entirely equal for patients with and without MB but was performed by a team of culturally sensitive therapists, who were trained to take into account cultural characteristics and their patients' history of migration.

Against this background, this study aims to examine whether there are differences regarding the depressive, somatoform, anxiety, and posttraumatic symptom severity between patients with and without MB at admission and discharge from inpatient psychotherapy; investigate the effectiveness of therapy regarding the above-mentioned symptoms in psychosomatic inpatient and outpatient clinics of migrant and non-migrant patients; and analyze the prevalence of diagnoses among the groups.

Based on the above-mentioned study results, we hypothesized that the symptom burden at admission would be higher and the effectiveness of treatment would be lower among migrant patients compared to non-migrant patients.

## Materials and Methods

### Design and Procedure

All patients starting psychotherapeutic inpatient treatment at the University Clinic of Psychosomatic Medicine and Psychotherapy in Erlangen and its affiliated psychosomatic department at the community hospital of Ebermannstadt were asked to participate in this study between October 2018 and October 2019. Patients treated in an outpatient clinic, which is part of the University Clinic in Erlangen, were also included in the study. Inclusion criteria covered the following: at least 18 years old, no acute psychotic disorder or acute suicidality, and sufficient German knowledge to understand and answer the questionnaires. Patients could be enrolled in the study at admission (T0) if the date of questionnaire completion did not go beyond the admission date by more than 10 days and if at least 50% of the questionnaires were filled out. Similarly, patients were enrolled at discharge (T1) if completion of the questionnaire and the discharge date did not differ by more than 10 days. We used self-administered questionnaires surveying symptom severity. We used paper-based questionnaires in the community hospital during the whole study period. In the other study settings, we started with digital data acquisition at the end of 2018. The questionnaires were handed out to the patients in the form of “paper-pencil” or tablets by the nursery staff at admission and discharge. The first author and an employee of the clinic controlled all steps, especially if informed consent was given. The treatment course of patients who did not want to take part in the study was not further pursued. For non-responder analysis, their gender and age were pseudonymously documented.

Treatment offered to the patients was the same in all clinics, including the outpatient clinic. The clinics under investigation follow an eclectic psychotherapeutic approach (in single and group therapy), including integrative depth-psychological and behavioral therapeutic concepts. In addition to psychotherapy, the clinics under investigation offered a therapy schedule which all patients followed equally during their treatment. The schedule included: psychoeducation, interaction groups, disorder-specific group therapy, concentrated movement therapy, art therapy, skills training (according to M. Linehan), mindfulness practice, and family sessions. If necessary, patients also received medical treatment. In the outpatient clinic, regular treatment duration was eight weeks, whereas in the inpatient clinics standard duration of treatment was at least eight weeks depending on the disorder but could vary. Patients received the survey within one week after admission (pre) and at least one week before discharge (post). International Statistical Classification of Diseases and Related Health Problems, 10th revision (ICD-10) coded diagnoses (F-diagnoses) were extracted from the therapists' letters at discharge. We used the definition of MB from the “Mikrozensus 2018” (1), a representative population-based survey run every year by the German National Institute of Statistics. It defines a person having MB if he/she or at least one of his/her parents did not obtain German citizenship by birth. This includes immigrant and nonimmigrant foreigners, immigrant and nonimmigrant naturalized persons, (late) emigrants (Spät-)Aussiedler, persons who obtained German citizenship through adoption, and German-born children from the above-mentioned groups. Displaced persons from former German regions (“Vertriebene”) or their children are not considered as persons with MB. Therapy offered to patients was culturally sensitive. The clinic's therapists are trained to include the patient´s history of migration in their anamnesis and emphasize migratory and cultural issues during therapy.

### Ethics Statement

The present study was approved by the Ethics Committee of the Medical Faculty of the Friedrich-Alexander University Erlangen-Nürnberg (FAU) (Project identification code: 232_14B). Written informed consent was obtained from all participants.

### Instruments

#### Patient Health Questionnaire: Somatization Module (PHQ-15)

The PHQ-15, part of the Patient Health Questionnaire (PHQ), a widely established screening instrument for common mental disorders, is a self-assessment instrument used to diagnose somatoform disorders and grade somatic complaints. In 13 items, the patient can answer questions rating how bothered he/she was during the last four weeks by common somatic symptoms on a scale of 0 (“not bothered at all”), 1 (“bothered a little”), and 2 (“bothered a lot”). Two further items (having little energy and trouble sleeping) coincide with the PHQ-9. The total score is 30 and cutoffs of 5, 10, and 15 points serve to differentiate mild, moderate, and severe symptom levels, respectively (30). In the validation study, Cronbach's α was 0.80 (31). In the German validation study, Cronbach's α was found to be 0.79 (32). In the present study, we obtained an internal consistency (Cronbach's α) of 0.81 at T0 and 0.82 at T1.

#### Patient Health Questionnaire: Depression Module (PHQ-9)

The PHQ-9 is part of the PHQ. The questionnaire is used to assess the severity of depressive symptoms and to categorize patients with major depression. It is aligned with nine main criteria to diagnose major depression (33) and has nine items that can be answered by the patient in self-assessment on a scale from 0 (not at all) to 3 (nearly every day). Scores of 0–4, 5–9, 10–14, 15–19, and 20–27 indicate minimal, mild, moderate, moderately severe, and severe depression, respectively (30). The validation study showed Cronbach's α = 0.89 (34). Cronbach's α in the German validation study was found to be 0.88 (32). In the present study, Cronbach's α was 0.84 at T0 and 0.88 at T1.

#### Patient Health Questionnaire: General Anxiety Disorder Module (GAD-7)

The GAD-7 is part of the PHQ. It consists of seven items and is commonly used to screen for general anxiety disorders based on criteria from the Diagnostic and Statistical Manual of Mental Disorders (DSM-IV). Similar to the PHQ-9, the patient is asked to assess on a scale from 0 (not at all) to 3 (almost every day) how often he/she had been bothered by seven common anxiety disorder symptoms during the last two weeks. Scores of 0–4, 5–9, 10–14, and 15–21 indicate minimal, mild, moderate, and severe anxiety symptoms, respectively (30). GAD-7 was shown to be a reliable and valid instrument with high internal consistency (Cronbach's α = 0.92) to screen for general anxiety disorder and estimate its severity (35). The German version shows a Cronbach's α of 0.89 (36). In the present study, Cronbach's α was 0.87 at T0 and 0.89 at T1.

#### Posttraumatic Stress Disorder Checklist (PCL-5)

The Posttraumatic Stress Disorder Checklist for DSM-V (PCL-5) is a self-reported questionnaire used to screen for PTSD. It consists of 20 items that ask for the most common symptoms on a scale from 0 (not at all) to 4 (extremely). The items are classified in four subscales that coincide with the four subscales from the DSM-V PTSD diagnosis: intrusion (items 1–5), avoidance (items 6–7), negative alterations in cognition and mood (items 8–14), and alterations in arousal and reactivity (items 15–20) (37). Summing up the 20 item scores, a value equal to or higher than 33 is necessary to diagnose PTSD (38). The authorized German version shows an internal consistency of α = 0.95 (38). In this study, the PCL-5 was used together with the Live Events Checklist for DSM-V (LEC-5) to find out how many and which traumatic events took place and how they were experienced. In the present study, Cronbach's α was 0.94 at T0 and 0.96 at T1.

### Statistical Analysis

Data analyses were conducted with SPSS V.26. All patients had filled out at least 50% of the questionnaires. After analyzing missing values, questionnaires with ≤20% missing values were completed with the expectation-maximization algorithm. Means, standard deviations, ranges, and frequencies were computed to profile the sociodemographic, migration-specific, and clinical characteristics of the total sample and subgroups. After applying exclusion criteria at discharge, all patients under treatment for ≥28 days who completed the questionnaire at discharge were defined as completers. Patients with less than 28 days of treatment and/or without a completed questionnaire at discharge were defined as non-completers. In order to minimize missing values, we applied the intention to treat (ITT) method (39), more specifically the last observation carried forward method, for all those who had not completed the survey at discharge. We performed all calculations at T1 for the full-completer sample and the sample that included the ITT sample. Since the results corresponded, we will only refer to the ITT sample. T-tests for dependent variables were used for pre-post comparisons, whereas t-tests for independent variables were used for intergroup comparisons. In case normal distribution was not given, nonparametric tests, such as Wilcoxon signed-rank rest for dependent and Mann–Whitney U-test for independent variables, were performed. To test for differences of categorical variables, chi-squared test was applied. In order to measure the effect size, we computed Cohen's d (40). Small effect sizes can be assumed if d > 0.2, medium effect sizes if d > 0.5, and large effect sizes if d > 0.8.

Furthermore, clinically significant changes were analyzed among the completer sample with the reliable change index (RCI) (41):

$$\text{RCI}=\frac{{\text{X}}_{\text{post}}\;-{\text{ X}}_{\text{pre}}}{{\text{S}}_{\text{diff}}},\;{\text{S}}_{\text{diff}}=\sqrt{2\times {\left({\text{S}}_{\text{E}}\right)}^{2}},\;{\text{S}}_{\text{E}}=\text{SD}\times \sqrt{1-{\text{r}}_{\text{xx}}}$$

where X_post_ is posttest value, X_pre_ is pretest value, S_diff_ is standard error of difference between the two test scores, SE is standard error of measurement, SD is standard deviation of the norm population, and r_xx_ is Cronbach's α.

Norm population data as well as Cronbach's α were drawn from Kocavelant et al. (42) for PHQ-15, from Hartung et al. (43) and Kocavelant et al. (42) for PHQ-9, and from Löwe et al. (36) for GAD-7. Norm population data for PCL-5 in Germany do not exist yet. Response to treatment was assumed when RCI was smaller than –1.96 (41). Remission after treatment was defined as response plus a post value of less than 10, since this is an established cutoff point for clinically significant symptoms on assessed scores (30). In all analyses, a significance level of p < 0.05 was determined. In order to equalize the conditions of the PHQ-15 questionnaire for differences in gender, we additionally calculated a sum score excluding the menstruation item (PHQ-15*).

## Results

### Sample Characteristics

**Figure 1:**
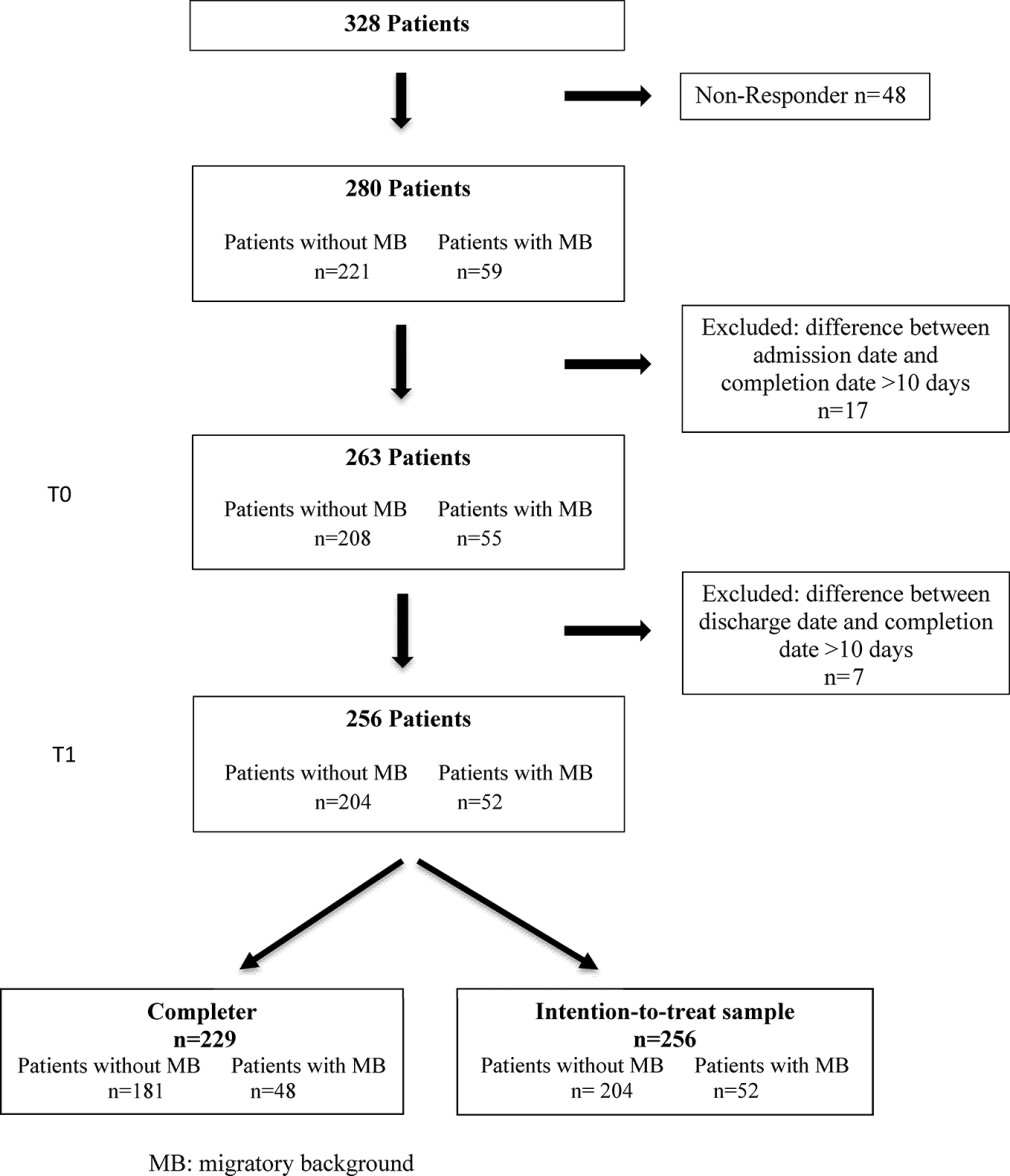
Flowchart.

Of the 328 patients who entered treatment in the period under investigation, all met basic inclusion criteria. In all, 280 gave their written consent, resulting in a response rate of 85.4%, and 17 out of the 280 study patients had to be excluded due to late completion of the questionnaire more than 10 days after admission (**Figure 1**).

#### Non-Responder Analysis

The non-responder analysis showed no significant differences between non-responders and responders in relation to age [non-responders: M = 37.6 years, SD = 14.0; responders: M = 39.3 years, SD = 13.4; t (326) = -0.802, p = 0.423] or gender [non-responders: men 37.5% (n = 18), women 62.5% (n = 30); responders: men 31.8% (n = 89), women 68.2% (n = 191); χ² (1) = 0.609, p = 0.435].

#### Non-Completer Analysis

Out of 256 patients at T1, 229 (89.5%) were completers and 27 (10.5%) were non-completers. Non-completers and completers did not show significant differences in relation to sociodemographic and migration-related variables (gender, age, family status, education, employment status, country of birth, migratory background), or symptom severity at admission. High comorbidity was not significantly associated with treatment dropout (Z = -1.587, p = 0.112).

#### Sociodemographic Data

Our study included 263 patients, 180 women (68.4%) and 83 men (31.6%); 208 (79.1%) patients were without MB, 139 women (66.8%) and 69 men (33.2%). The study sample consisted of 55 (20.9%) patients with MB, 41 women (74.5%) and 14 men (25.5%). The average age of sample participants was 39.3 years (SD = 13.3). Most (n = 138, 52.5%) were single, 47.1% (n = 124) lived together with a partner, 53.6% (n = 141) finished middle school, and 37.6% (n = 99) worked full time (**Table 1**). There were no striking differences between the two groups (patients with and without MB) regarding the sociodemographic variables gender, age, civil status, education, employment status, and pretreatment at baseline.

**Table 1:** Sociodemographic data of the total sample and patients with and without migratory background.

	Total sample (N=263)	Patients without MB (n=208)	Patients with MB (n=55)	Patients with MB 1^st^ Generation (n=26)	Patients with MB 2^nd^ Generation (n=29)
**Gender, n (%)**					
Women	180 (68.4)	139 (66.8)	41 (74.5)	20 (76.9)	21 (72.4)
Men	83 (31.6)	69 (33.2)	14 (25.5)	6 (23.1)	8 (27.6)
**Age (years)**					
M (SD)	39.3 (13.3)	39.3 (13.7)	39.5 (11.7)	44.5 (8.9)	35.0 (12.3)
Range	18–82	18–82	18–62	19–58	18–62
**Age group, n (%)**					
<25	41 (15.6)	32 (15.4)	9 (16.4)	1 (3.8)	8 (27.6)
25-34	64 (24.3)	54 (26.0)	10 (18.2)	3 (11.5)	7 (24.1)
35-44	61 (23.2)	46 (22.1)	15 (27.3)	7 (26.9)	8 (27.6)
45-54	56 (21.3)	40 (19.2)	16 (29.1)	12 (46.2)	4 (13.8)
55-64	38 (14.4)	33 (15.9)	5 (9.1)	3 (11.5)	2 (6.9)
≥65	3 (1.1)	3 (1.4)	–	–	–
**Family status, n (%)**					
Single	138 (52.5)	115 (55.3)	23 (41.8)	5 (19.2)	18 (62.1)
Married/registered partners	90 (34.2)	65 (31.3)	25 (45.5)	17 (65.4)	8 (27.6)
Separated/divorced	29 (11.0)	24 (11.5)	5 (9.1)	3 (11.5)	2 (6.9)
Widowed	3 (1.1)	2 (1.0)	1 (1.8)	–	1 (3.4)
No data	3 (1.1)	2 (1.0)	1 (1.8)	1 (3.8)	–
**Living together with partner, n (%)**					
Yes	124 (47.1)	96 (46.2)	28 (50.9)	16 (61.5)	12 (41.4)
No	134 (51.0)	107 (51.4)	27 (49.1)	10 (38.5)	17 (58.6)
No data	5 (1.9)	5 (2.4)	–	–	–
**Education, n (%)**					
No educational certificate	8 (3.0)	7 (3.4)	1 (1.8)	–	1 (3.4)
Primary School	9 (3.4)	7 (3.4)	2 (3.6)	1 (3.8)	1 (3.4)
Middle School	141 (53.6)	112 (53.8)	29 (52.7)	12 (46.2)	17 (58.6)
Secondary/Vocational School	89 (33.8)	71 (34.1)	18 (32.7)	10 (38.5)	8 (27.6)
University degree	10 (3.8)	8 (3.8)	2 (3.6)	1 (3.8)	1 (3.4)
Other	3 (1.1)	1 (0.5)	2 (3.6)	2 (7.7)	–
No data	3 (1.1)	2 (1.0)	1 (1.8)	–	1 (3.4)
**Employment status, n (%)**					
Employed full-time	99 (37.6)	81 (38.9)	18 (32.7)	11 (42.3)	7 (24.1)
Employed part-time	52 (19.8)	37 (17.8)	15 (27.3)	8 (30.8)	7 (24.1)
Unemployed	13 (4.9)	10 (4.8)	3 (5.5)	1 (3.8)	2 (6.9)
Job-seeking	26 (9.9)	19 (9.1)	7 (12.7)	3 (11.5)	4 (13.8)
Pensioner	12 (4.6)	12 (5.8)	–	–	–
Pension because of a reduction in earning capacity	7 (2.7)	6 (2.9)	1 (1.8)	1 (3.8)	–
Trainee/Student	23 (8.7)	19 (9.1)	4 (7.3)	1 (3.8)	3 (10.3)
Sick leave	9 (3.4)	7 (3.4)	2 (3.6)	1 (3.8)	1 (3.4)
Other	18 (6.8)	15 (7.2)	3 (5.5)	–	3 (10.3)
No data	4 (1.5)	2 (1.0)	2 (3.6)	–	2 (6.9)
**Duration of treatment (days)**					
M (SD)	55.3 (16.5)	55.3 (18.0)	55.3 (8.2)	52.2 (8.9)	58.1 (6.4)
Range	1-183	1-183	35-77	35-77	52-72
Number of pretreatments*, n (%)					
None	126 (47.9)	96 (46.2)	30 (54.5)	16 (61.5)	14 (48.3)
1-2	91 (34.6)	73 (35.1)	18 (32.7)	8 (30.8)	10 (34.5)
3-5	32 (12.2)	28 (13.5)	4 (7.3)	1 (3.8)	3 (10.3)
6-10	10 (3.8)	8 (3.8)	2 (3.6)	–	2 (6.9)
11-19	2 (0.8)	1 (0.5)	1 (1.8)	1 (3.8)	–
>20	1 (0.4)	1 (0.5)	–	–	–
No data	1 (0.4)	1 (0.5)	–	–	–

MB, Migratory background, *: hospital/daily hospital psychosomatic/psychiatric treatments.

#### Migration-Related Data

In the study sample, 55 patients (20.9%) had MB. Of these 55 patients, 26 (47.3%) lived in Germany in the first generation, whereas 29 (52.7%) lived in Germany in the second generation. Migrant patients had 28 different countries of origin. The most frequent countries of origin were Poland (n = 10, 18.2%), followed by Turkey (n = 7, 12.7%), Italy, Romania, Ukraine, and Hungary (n = 3, 5.5%). Further information about the migration status is shown in **Table 2**.

**Table 2:** Migration-related data of the total migratory sample and first and second generation.

	Patients with MB (N=55)	First Generation (n=26)	Second Generation (n=29)
**Country of birth, n (%)**			
Germany	29 (52.7)	–	29 (100)
Other country	26 (47.3)	26 (100)	–
**Length of residence in Germany (years)**			
M (SD	–	27.2 (10.2)	–
**Range**	–	8-49	–
**Length of residence in Germany (years), n (%)**			
<10	–	2 (7.7)	–
10-19	–	3 (11.5)	–
20-29	–	12 (46.2)	–
30-39	–	4 (15.4)	–
≥40	–	4 (15.4)	–
No data	–	1 (3.8)	–
**Age at immigration (years)**			
M (SD)	–	16.9 (10.3)	–
Range	–	1-35	–
**Age at immigration (years), n (%)**			
<10	–	8 (30.8)	–
10-20	–	7 (26.9)	–
21-35	–	10 (38.5)	–
No data	–	1 (3.8)	
**Legal Status, n (%)**			
German citizenship	42 (76.4)	19 (73.1)	23 (79.3)
Double Citizenship	1 (1.8)	1 (3.8)	–
Unlimited residence permit	5 (9.1)	2 (7.7)	3 (10.3)
Other Citizenship	6 (10.9)	4 (15.4)	2 (6.9)
Limited residence permit	1 (1.8)	–	1 (3.4)
**Language proficiency**, **n (%)**			
German as mother tongue	23 (41.8)	4 (15.4)	19 (65.5)
Very good	12 (21.8)	7 (26.9)	5 (17.2)
Good	16 (29.1)	12 (46.2)	4 (13.8)
Moderate	4 (7.3)	3 (11.5)	1 (3.4)
Bad	–	–	–
Very bad	–	–	–

MB, Migratory background.

#### Clinical Data

A very high percentage (87.1%) of the sample population had more than one mental and behavioral disorder (F-diagnosis from ICD-10): 41.4% (n = 109) of the patients had two F-diagnoses, and 30.4% (n = 80) had three to six (**Table 3**). The most frequent main diagnoses were neurotic, stress-related, and somatoform disorders (F4) (48.3%, n = 127), affective disorders (F3) (29.7%, n = 78), and eating disorders (F5) (16.7%, n = 44). The distribution of the main diagnoses in our sample differed significantly between the two analyzed groups [χ² (3) = 8.398, p = 0.038]. Affective disorders (31.3%, n = 65 vs. 23.6%, n = 13) and eating disorders (19.2%, n = 40 vs. 7.3%, n = 4) were more frequent among patients without MB, whereas neurotic, stress-related, and somatoform disorders (60%, n =33 vs. 45.2%, n = 94) as well as disorders of adult personality and behavior (9.1%, n = 5 vs. 4.3%, n = 9) were more frequent among patients with MB. Especially F5 diagnoses were associated with patients without MB, while F4 diagnoses were more frequently associated with MB. A more detailed distribution of diagnoses is presented in **Table 4**. The median duration of treatment was 55.3 days (SD = 16.5, range 1 to 183 days). Almost half of all patients (n = 126, 47.9%) had not undergone any outpatient or inpatient psychosomatic or psychiatric treatment before (**Table 1**).

**Table 3:** Number of ICD-10 coded mental and behavioral disorders of the total sample and subdivided by migratory background.

	Total Sample (N=263)	Patients without MB (n=208)	Patients with MB (n=55)
**Number of ICD-10 coded mental and behavioral disorders, n (%)**			
one	34 (12.9)	24 (11.5)	10 (18.2)
two	109 (41.4)	89 (42.8)	20 (36.4)
three	80 (30.4)	62 (29.8)	18 (32.7)
four	33 (12.5)	26 (12.5)	7 (12.7)
five	5 (1.9)	5 (2.4)	–
six	2 (0.8)	2 (1.0)	–
**Number of ICD-10 coded mental and behavioral disorders, n (%)**			
F3	173 (65.8)	132 (63.5)	41 (74.5)
F4	109 (41.4)	86 (41.3)	23 (41.8)
F5	9 (3.4)	8 (3.8)	1 (1.8)
F6	35 (13.3)	31 (14.9)	4 (7.3)
Others	33 (12.5)	29 (13.9)	4 (7.3)

MB, Migratory background; ICD-10, International classification of diseases and related health conditions; F3: F30-F39 affective disorders; F4: F40-F48 neurotic, stress-related, and somatoform disorders; F5: F50.1-9 eating disorders; F6: F60-F69 disorders of adult personality and behavior.

**Table 4:** ICD-10 coded main psychiatric diagnoses of the total sample and subdivided by migratory background and gender.

	Total sample (N=263)	Women (n=180)	Men (n=83)	Patients without MB (n=208)	Women (n=139)	Men (n=69)	Patients with MB (n=55)	Women (n=41)	Men (n=14)
**Main ICD-10 coded psychiatric diagnoses**, **n (%)**									
**F3**	**78 (29.7)**	**50 (27.8)**	**28 (33.7)**	**65 (31.3)**	**43 (30.9)**	**22 (31.9)**	**13 (23.6)**	**7 (17.1)**	**6 (42.9)**
F32.1	18 (6.8)	12 (6.7)	6 (7.2)	14 (6.7)	9 (6.5)	5 (7.2)	4 (7.3)	3 (7.3)	1 (7.1)
F32.2	1 (0.4)	1 (0.6)	–	1 (0.5)	1 (0.7)	–	–	–	–
F32.3	1 (0.4)	1 (0.6)	–	1 (0.5)	1 (0.7)	–	–	–	–
F33.1	56 (21.3)	36 (20.0)	20 (24.1)	49 (23.6)	32 (23.0)	17 (24.6)	7 (12.7)	4 (9.8)	3 (21.4)
F33.2	2 (0.8)	–	2 (2.4)	–	–	–	2 (3.6)	–	2 (14.3)
**F4**	**127 (48.3)**	**81 (45.0)**	**46 (55.4)**	**94 (45.2)**	**55 (39.6)**	**39 (56.5)**	**33 (60.0)**	**26 (63.4)**	**7 (50.0)**
F40	4 (1.5)	2 (1.1)	2 (2.4)	4 (1.9)	2 (1.4)	2 (2.9)	–	–	–
F40.1	11 (4.2)	6 (3.3)	5 (6.0)	10 (4.8)	6 (4.3)	4 (5.8)	1 (1.8)	–	1 (7.1)
F41	9 (3.4)	6 (3.3)	3 (3.6)	6 (2.9)	3 (2.2)	3 (4.3)	3 (5.5)	3 (7.3)	–
F42	12 (4.6)	6 (3.3)	6 (7.2)	9 (4.3)	4 (2.9)	5 (7.2)	3 (5.5)	2 (4.9)	1 (7.1)
F43	10 (3.8)	7 (3.9)	3 (3.6)	7 (3.4)	4 (2.9)	3 (4.3)	3 (5.5)	3 (7.3)	–
F43.1	46 (17.5)	35 (19,4)	11 (13.3)	31 (14.9)	22 (15.8)	9 (13.0)	15 (27.3)	13 (31.7)	2 (14.3)
F44	2 (0.8)	2 (1.1)	–	2 (1.0)	2 (1.4)	–	–	–	–
F45	33 (12.5)	17 (9.4)	16 (19.3)	25 (12.0)	12 (8.6)	13 (18.8)	8 (14.5)	5 (12.2)	3 (21.4)
**F5**	**44 (16.7)**	**39 (21.7)**	**5 (6.0)**	**40 (19.2)**	**35 (25.2)**	**5 (7.2)**	**4 (7.3)**	**4 (9.8)**	–
F50	15 (5.7)	14 (7.8)	1 (1.2)	14 (6.7)	13 (9.4)	1 (1.4)	1 (1.8)	1 (2.4)	–
F50.1	8 (3.0)	7 (3.9)	1 (1.2)	7 (3.4)	6 (4.3)	1 (1.4)	1 (1.8)	1 (2.4)	–
F50.2	8 (3.0)	8 (4.4)	–	7 (3.4)	7 (5.0)	–	1 (1.8)	1 (2.4)	–
F50.9	13 (4.9)	10 (5.6)	3 (3.6)	12 (5.8)	9 (6.5)	3 (4.3)	1 (1.8)	1 (2.4)	–
**F6**	**14 (5.3)**	**10 (5.6)**	**4 (4.8)**	**9 (4.3)**	**6 (4.3)**	**3 (4.3)**	**5 (9.1)**	**4 (9.8)**	**1 (7.1)**
F60-F62	12 (4.6)	8 (4.4)	4 (4.8)	7 (3.4)	4 (2.9)	3 (4.3)	5 (9.1)	4 (9.8)	1 (7.1)
F63-F69	2 (0.8)	2 (1.1)	–	2 (1.0)	2 (1.4)	–	–	–	–

MB, Migratory background; ICD-10, International classification of diseases and related health conditions; F3: F30-F39 affective disorders; F32.1: moderate depressive episode; F32.2: severe depressive episode without psychotic symptoms; F32.3: severe depressive episode with psychotic symptoms; F33.1: recurrent depressive disorder, current episode moderate; F33.2: recurrent depressive disorder, current episode severe without psychotic symptoms; F4: F40-F48 neurotic, stress-related, and somatoform disorders; F40: phobic anxiety disorders; F40.1: social phobias; F41: other anxiety disorders; F42: obsessive-compulsive disorder; F43: reaction to severe stress, and adjustment disorders; F43.1: post-traumatic stress disorder; F44: dissociative [conversion] disorders; F45: somatoform disorders; F5: F50-F59 behavioral syndromes associated with physiological disturbances and physical factors; F50: eating disorders; F50.1: atypical anorexia nervosa; F50.2: bulimia nervosa; F50.9: eating disorder, unspecified; F6: F60-F69 disorders of adult personality and behavior; F60-F62: specific personality disorders, mixed and other personality disorders, enduring personality changes, not attributable to brain damage and disease; F63-F69: habit and impulse disorders, gender identity disorders, disorders of sexual preference, psychological and behavioral disorders associated with sexual development and orientation, other disorders of adult personality and behavior, unspecified disorder of adult personality and behavior.

### Outcome Measures (Total Sample and Comparison of Patients With and Without MB)

#### Baseline

At admission, the symptom burden of the total sample was moderate to moderately severe on the somatization, depression, and anxiety scales (PHQ-15: M = 12.9, SD = 5.6; PHQ-9: M = 14.4, SD = 5.7; GAD-7: M = 11.3, SD = 5.1). The average score for posttraumatic symptoms (PCL-5) was M = 33.3 (SD = 18.7) out of 80. In our sample, migrant patients indicated at least one trauma on the Live Events Checklist for DSM-V in 96.4% (n = 53) of the cases, and nonmigrant patients indicated at least one in 91.8% (n = 191). Patients with MB had an overall higher symptom burden at admission. The two groups significantly differed in symptom severity at admission concerning somatization (p = 0.025, d = 0.345) and posttraumatic symptoms (p = 0.008, d = 0.424). This result remained statistically significant when excluding the menstruation item. More information is presented in **Table 5**. Data concerning outcome measures at baseline and discharge are also presented for the full-completer sample in **Table 6**.

**Table 5:** Symptoms at admission and discharge of the total intention-to-treat-sample and patients with and without migratory background in comparison.

	T0						T1					
	Total sample (N=263) M (SD)	Patients without MB (N=208) M (SD)	Patients with MB (N=55) M (SD)	Comparison			Total sample (N=256) M (SD)	Patients without MB (N=204) M (SD)	Patients with MB (N=52) M (SD)	Comparison		
				**T**	**p**	**d**				**T**	**p**	**d**
**PHQ-15**	12.9 (5.6)	12.5 (5.6)	14.5 (5.7)	-2.254	**0.025**	**0.345**	11.4 (5.4)	10.9 (5.1)	13.5 (6.0)	-3.258	**0.001**	**0.507**
PHQ-15*	12.5 (5.4)	12.2 (5.4)	13.9 (5.4)	-2.118	**0.035**	**0.324**	11.0 (5.1)	10.5 (4.9)	13.0 (5.6)	-3.077	**0.002**	**0.478**
**PHQ-9**	14.4 (5.7)	14.1 (5.8)	15.5 (5.3)	-1.568	0.118	0.238	10.4 (5.7)	10.1 (5.8)	11.8 (5.4)	-2.014	**0.045**	**0.313**
**GAD-7**	11.3 (5.1)	11.0 (5.2)	12.1 (4.5)	-1.224	0.222	0.208	8.2 (4.9)	7.8 (4.9)	9.9 (4.7)	-2.527	**0.012**	**0.428**
**PCL-5**	33.3 (18.7)	31.6 (18.3)	39.4 (19.2)	-2.679	**0.008**	**0.424**	29.4 (19.8)	28.0 (19.4)	34.5 (20.4)	-2.062	**0.040**	**0.329**

MB, migratory background; PHQ-15, Patient Health Questionnaire Somatization Module;* without Menstruation Item; PHQ-9, Patient Health Questionnaire Depression Module; GAD-7, General Anxiety Disorder Short Scale; PCL-5, PTSD-Checklist for DSM-5; Varying sample sizes; significant p-values and the corresponding effect sizes are marked in bold.

**Table 6:** Symptoms at admission and discharge of the total completer sample and patients with and without migratory background in comparison.

	T0						T1					
	Total sample (N=263) M (SD)	Patients without MB (N=208) M (SD)	Patients with MB (N=55) M (SD)	Comparison			Total sample (N=229) M (SD)	Patients without MB (N=181) M (SD)	Patients with MB (N=48) M (SD)	Comparison		
				T	p	d				T	p	d
**PHQ-15**	12.9 (5.6)	12.5 (5.6)	14.5 (5.7)	-2.254	**0.025**	**0.345**	11.1 (5.3)	10.5 (4.9)	13.5 (6.1)	-3.548	**<0.001**	**0.577**
PHQ-15*	12.5 (5.4)	12.2 (5.4)	13.9 (5.4)	-2.118	**0.035**	**0.324**	10.8 (5.1)	10.2 (4.8)	12.9 (5.7)	-3.375	**0.001**	**0.549**
**PHQ-9**	14.4 (5.7)	14.1 (5.8)	15.5 (5.3)	-1.568	0.118	0.238	9.9 (5.5)	9.5 (5.6)	11.3 (5.0)	-2.043	**0.042**	**0.332**
**GAD-7**	11.3 (5.1)	11.0 (5.2)	12.1 (4.5)	-1.224	0.222	0.208	7.8 (4.7)	7.3 (4.5)	9.7 (4.6)	-3.068	**0.002**	**0.535**
**PCL-5**	33.3 (18.7)	31.6 (18.3)	39.4 (19.2)	-2.679	**0.008**	**0.424**	28.9 (19.7)	27.4 (19.4)	34.0 (20.1)	-2.050	**0.042**	**0.339**

MB, migratory background; PHQ-15, Patient Health Questionnaire Somatization Module;* without Menstruation Item; PHQ-9, Patient Health Questionnaire Depression Module; GAD-7, General Anxiety Disorder Short Scale; PCL-5, PTSD-Checklist for DSM-5; varying sample sizes; significant p-values and the corresponding effect sizes are marked in bold.

#### Discharge

At discharge, patients with and without MB had fewer symptoms than at admission. However, the two groups differed significantly by severity of symptoms at discharge on all scales (somatization: p = 0.001, d = 0.507; depression: p = 0.045, d = 0.313; anxiety: p = 0.012, d = 0.428; posttraumatic symptoms: p = 0.040, d = 0.329). More detailed results are presented in **Tables 5** and **6**.

#### Symptom Improvement

Comparing the symptoms at admission and discharge, we show that the symptom burden of patients without MB significantly improved on all scales (somatization: p < 0.001, d = -0.304; depression: p < 0.001, d = -0.692; anxiety: p < 0.001, d = -0.605; posttraumatic symptoms: p < 0.001, d = -0.204). Patients with MB improved significantly on the depression and anxiety scale but could not significantly alleviate somatization and posttraumatic symptoms (depression: p < 0.001, d = -0.649; anxiety: p = 0.002, d = -0.441) (**Table 7**). Comparing the two scales that changed significantly for both groups, depression and anxiety, effect sizes were higher among non-migrant patients (PHQ-9: d = -0.692 vs. -0.649; GAD-7: d = -0.605 vs. -0.441).

**Table 7:** Symptom change of the total intention-to-treat-sample and patients with and without migratory background.

	Total sample T0-T1			Patients without MB T0-T1			Patients with MB T0-T1		
	T	p	d	T	p	d	T	p	d
**PHQ-15**	5.892	**<0.001**	**-0.264**	5.957	**<0.001**	**-0.304**	1.401	0.167	-0.138
PHQ-15 *	5.998	**<0.001**	**-0.274**	5.949	**<0.001**	**-0.311**	1.572	0.122	-0.155
**PHQ-9**	12.217	**<0.001**	**-0.681**	11.114	**<0.001**	**-0.692**	5.056	**<0.001**	**-0.649**
**GAD-7**	9.136	**<0.001**	**-0.573**	8.560	**<0.001**	**-0.605**	3.246	**0.002**	**-0.441**
**PCL-5**	3.951	**<0.001**	**-0.190**	3.951	**<0.001**	**-0.204**	1.225	0.227	-0.152

MB, migratory background; PHQ-15, Patient Health Questionnaire Somatization Module;* without Menstruation Item; PHQ-9, Patient Health Questionnaire Depression Module; GAD-7, General Anxiety Disorder Short Scale; PCL-5, PTSD-Checklist for DSM-5; varying sample sizes; significant p-values and the corresponding effect sizes are marked in bold.

Among the full-completer sample, we obtained a response of 23.8% for somatoform symptoms, 54.8% for depressive symptoms, and 44.3% for anxiety symptoms. Full remission of somatoform symptoms was achieved by 12.6%, depressive symptoms by 36.0%, and anxiety symptoms by 35.1%. Migrant patients showed slightly lower rates regarding response and remission for PHQ-15 and GAD-7. For PHQ-9, patients with MB had a somewhat higher response but slightly lower remission than non-migrant patients. Findings concerning symptom improvement are also presented for the full-completer sample in **Table 8**.

**Table 8:** Symptom change of the total completer sample and patients with and without migratory background.

	Total sample T0-T1					Patients without MB T0-T1					Patients with MB T0-T1				
	T	p	d	Resp*	Rem**	T	p	d	Resp*	Rem**	T	p	d	Resp*	Rem**
**PHQ-15**	5.818	**<0.001**	**-0.287**	23.8%	12.6%	5.888	**<0.001**	**-0.337**	24.4%	13.1%	1.404	0.167	-0.148	21.3%	10.6%
PHQ-15 *	5.936	**<0.001**	**-0.299**	–	–	5.888	**<0.001**	**-0.345**	–	–	1.575	0.122	-0.165	–	–
**PHQ-9**	12.447	**<0.001**	**-0.759**	54.8%	36.0%	11.324	**<0.001**	**-0.771**	53.9%	37.2%	5.161	**<0.001**	**-0.727**	58.3%	31.3%
**GAD-7**	9.187	**<0.001**	**-0.638**	44.3%	35.1%	8.621	**<0.001**	**-0.687**	46.2%	37.2%	3.267	**0.002**	**-0.464**	36.8%	26.3%
**PCL-5**	3.834	**<0.001**	**-0.205**	–	–	3.814	**<0.001**	**-0.220**	–	–	1.228	0.226	-0.165	–	–

MB, Migratory background; PHQ-15, Patient Health Questionnaire Somatization Module; * without Menstruation Item; PHQ-9, Patient Health Questionnaire Depression Module; GAD-7, General Anxiety Disorder Short Scale; PCL-5, PTSD-Checklist for DSM-5; Resp*, Response: RCI <-1.96; Rem**: Remission RCI <-1.96 and post value <10; varying sample sizes; significant p-values and the corresponding effect sizes are marked in bold.

## Discussion

The aim of this study was to examine the effectiveness of inpatient psychotherapy among patients with and without MB. We analyzed symptom burden at admission and discharge, calculated the effects of inpatient psychotherapy, and demonstrated the distribution of diagnoses.

### Study Sample

Concerning gender distribution, this study sample confirmed the tendency of a larger proportion of female patients being in psychosomatic treatment programs (Steffanowski et al. (3): 56 studies, 64% women on average). Every fifth patient in the study sample had MB (20.9%). Compared to the proportion among the general population in Germany (25.5%) (1), they were slightly underrepresented. This result is partially in line with several studies on mental health care utilization among migrants, which identify underrepresentation of migrant patients in psychotherapeutic hospital settings and overrepresentation in psychiatric emergency care, forensic psychiatry, and departments of addiction (44, 45). Opposite results (11, 20, 46) showed that the proportion of migrant patients in psychiatric or psychotherapeutic inpatient care generally corresponds to the proportion of migrants in the population [22.3% (11), 17.4% (20), 17% (46)]. It can be assumed that our sample of patients with MB is not entirely representative of the very heterogeneous population of immigrants in Germany. Among them, 23 (41.8%) indicated German as their first language and none indicated bad or very bad German language proficiency. Taking into account that language is considered to be the main indicator for successful integration, most of the patients might be regarded as well integrated into the mainstream society. The majority had immigrated at a young age (M = 16.9 years, SD = 10.3) and the average duration of residence in Germany was 27.2 years (SD = 10.2).

### Main Results

Patients with MB had a significantly higher symptom burden regarding somatoform and posttraumatic symptoms at the beginning of treatment. The main diagnoses in our sample did differ significantly between the two analyzed groups. Within the F4 diagnosis group, especially F43.1 (PTSD) was twice as frequent among patients with MB. Patients with MB were more often burdened with neurotic, stress-related, and somatoform disorders (F4) and less often with affective disorders than non-migrant patients. Non-migrant patients had significantly improved symptom severity across all scales. Depression and general anxiety reached the highest effect sizes. Patients with MB also improved regarding all symptoms but only the improvements in depression and anxiety symptoms showed statistical significance.

### Severity at Baseline

The observation that patients with MB have an overall higher symptom burden corresponds with the results of earlier studies. Mösko et al. (10) showed that patients with MB from a psychosomatic rehabilitation hospital had significantly higher symptom severity of depression, somatization, and anxiety. Using a psychotherapeutic inpatient sample, Wiborg et al. (11) demonstrated that MB was associated with more symptoms at baseline based on the Symptom Checklist 90 (SCL-90). In outpatient facilities, several studies have also shown a higher symptom burden among migrant patients. In an outpatient sample, Erim et al. (19) detected a higher symptom burden at admission for posttraumatic symptoms among Turkish patients; however, somatization did not differ significantly from German reference values in that study. With regard to a non clinical sample Morawa et al. (47) detected higher levels of somatization among Turkish migrants compared to the German general population. Leidinger et al. (48) further demonstrated an overall higher symptom burden for depression, posttraumatic symptoms, somatization, and anxiety in Iranian patients from an outpatient doctor's office compared to German patients.

### Symptom Improvement

All patient's symptoms improved significantly on the assessed scales. Effect sizes varied from small (PHQ-15 and PCL-5) to medium (PHQ-9 and GAD-7). This finding aligns with current knowledge about the effectiveness of inpatient psychotherapy. Steffanowski et al. (3) showed that average effect sizes (Cohen's d) in inpatient psychosomatic rehabilitation are medium to high. Liebherz and Rabung (4, 5) conducted two meta-analyses examining the effectiveness of psychotherapeutic hospital treatment in Germany, including rehabilitation hospitals. They calculated an overall effect size of g = 0.71. As rehabilitation usually follows regular inpatient treatment, it can be assumed that our sample population was more burdened than sample populations from rehabilitation settings. This is also reflected in the number of psychiatric comorbidities in our sample. Mösko et al. (10) showed small effect sizes for inpatient psychosomatic rehabilitation treatment for depression, somatization, and anxiety. This trend is partially confirmed (regarding somatization) in our study by response and remission rates with the reliable change index (RCI). We could show that somatoform symptoms had smaller response and remission with psychotherapy than depressive and anxiety symptoms. More than one-third of the sample population reached full remission in relation to anxiety and depression, but only 12.6% reached remission related to somatization.

With reference to the therapy outcomes of patients with MB, our results partially contradict earlier studies. Mösko et al. (10), Göbber et al. (12), Brause et al. (13), and Zollmann et al. (16) concluded that patients with Turkish MB had worse therapy outcomes than patients without Turkish MB. We cannot fully confirm these findings. The group of Turkish migrants was too small (n = 7) to calculate their specific therapy outcomes. However, the heterogeneous group of migrant patients from our sample showed moderate therapy outcomes for somatization (PHQ-15) and posttraumatic symptoms (PCL-5) but significant improvement for depression (PHQ-9) and anxiety (GAD-7). The finding by Wiborg et al. (11) that patients with direct migratory experience improved more than other patients cannot be confirmed. Taking into account that the two groups (patients with and without MB) differed significantly on the PHQ-15 and PCL-5 scales at admission, it seems plausible that this difference remains stable along the therapeutic process. Since patients with MB were more burdened in relation to these symptoms at study initiation, it might have been more difficult for them to benefit from the treatment to the same extent as non-migrant patients. The tendency to experience less improvement of somatization and posttraumatic symptoms was also found among patients without MB. Although significant changes were achieved, effect sizes regarding the PHQ-15 and PCL-5 were only small (PHQ-15: d = -0.304; PCL-5: d = -0.204). It remains to be examined why migrant patients profit less from psychotherapy concerning somatization and traumatization. Reasons for this difference might be found in different sociocultural characteristics, disease concepts, and coping strategies or result from different traumatic experiences.

### Diagnoses

The higher prevalence of F4 diagnoses among patients with MB is consistent with current research. Schmeling-Klaudas et al., Erim et al., and Aragona et al. (14, 19, 49) demonstrated a high prevalence of somatoform disorders among migrant patients. Leidinger et al. (48) showed a significantly higher prevalence of PTSD but also of affective disorders in Iranian patients from a psychiatrist's office than in nonmigrant patients. Göbber et al. (12) also indicated more somatoform and affective disorders among migrant patients originating from Turkey than among German patients. A study examining the prevalence of mental disorders in a representative population sample could not find any significant differences in prevalence or severity of depression, anxiety, somatoform disorders, or PTSD between patients with and without MB (50). Yet, the authors stated that migrant patients had undergone significantly more traumatizing events in their lives. Similarly, in our sample, more migrant patients indicated at least one trauma in the Live Events Checklist for DSM-V than non-migrant patients. Bermejo et al. (51) conducted a cross-sectional analysis in which they could not confirm that migrants generally suffered from somatoform disorders more frequently. However, they showed significantly higher levels of somatization among migrants originating from Turkey and the former Soviet Union.

It is known that migration goes along with specific stressors such as, e.g., cultural adjustment processes, acquisition of a new language, separation from the family, and manifest or latent discrimination. At the same time, it is important to differentiate among the reasons for migration. Lindert et al., for example, demonstrated a prevalence of depression among refugees twice as high as that among labor migrants in the USA (52). First-generation labor migrants did not differ significantly in depression, anxiety, or PTSD prevalence from the native population. Although we did not concretely document the reasons for migration in our sample, taking into account the country of origin and the year of migration of the participants, it seems likely that the majority were labor migrants or their descendants. In order to distinguish between migratory stressors and to investigate whether these have any association with the individual psychological problems of the patient, it is important to make the MB a subject of discussion during therapy.

Another important factor for therapy outcome is the motivation of the patient toward psychotherapy. Some studies have analyzed atittudes toward psychotherapy among different migration groups (53–57). Bretz et al. (53), Calliess et al. (55), and Reich et al. (56) came to the conclusion that migrants with a Turkish background had a more negative attitude toward psychotherapy than nonmigrants. Ditte et al. (54) compared the attitudes of migrants with a Russian background and nonmigrants in Germany. Russian migrants in Germany also showed a more negative attitude toward psychotherapy than nonmigrants. This might be due to limited reputation and acceptance of psychotherapy in the countries of origin. In Western societies, the aims and methods of psychotherapy may follow an individualistic orientation of the society. On the contrary, many of the origin countries of migrants tend to show a more collectivist cultural orientation (58). Several studies have pointed out that illness beliefs vary widely among different cultures (59–62). Such culture-specific factors may interfere with the treatment outcomes of migrant patients. Although we did not collect data on the motivation for psychotherapy or illness beliefs, it is possible that among our sample these factors did partially contribute to worse treatment outcomes among patients with MB.

Qureshi and Collazo (25) stated that a variety of culture- and race-related factors might lead to a lower quality of mental health services for migrants. Apart from training for clinical psychotherapists on the cultural needs of migrant patients, no special adaption of therapy was undertaken for patients with MB in the departments participating in the study. Optimization of the therapy outcome may be reached when specialized culture-specific methods are implemented.

### Limitations and Strengths

The primary strength of the present study is the prospective study design, which made it possible to examine symptom improvement. The present study sample represents a realistic clinical example, hence our results reflect real clinical changes.

The diagnoses used in the study were thoroughly determined by the treating therapists during the time of treatment of each patient. Yet, in future studies, a standardized diagnostic interview might lead to more validity. Despite the challenges given by a clinical environment, we tried to implement a maximally standardized process. This was limited by variations both in the time patients needed to complete the surveys and in the duration of treatment. Furthermore, symptom scores are based on self-administered instruments and not on clinical interviews. Since we did not carry out a catamnestic analysis, it is possible that symptom ratings at discharge were higher than would be expected, as immediately before discharge, symptoms tend to increase shortly. As with many studies in psychotherapeutic research, we did not use a controlled study design, mainly for ethical reasons. This is why we cannot eliminate the time effect, which would suppose that some patients might also recover or show symptom improvement without treatment. Consequently, the validity of our results is limited. In order to approach the issue of missing control groups, a study was conducted in which the conclusion was drawn that psychiatric patients without any specific treatment had small effect sizes in symptom change (Cohen's d = 0.12) (63). This value can be used as a reference for a potential control group. The study was conducted in German, hence it was prone to selection bias, as only migrant patients with sufficient German proficiency were included. Finally, our investigation cannot explain why patients with migratory background benefited less concerning somatization and traumatization, as we have not examined important potential influencing factors. Accordingly, variables of interest for further studies on this topic could be among others: disease and health concepts, coping strategies, social support, resilience, acculturation strategies as well as pre- and postmigration risk factors (e.g. traumatic experiences, discrimination).

## Conclusions

This study shows that inpatient psychotherapy is effective. Yet, compared to patients without MB, the outcome of treatment for patients with MB is not as effective. While they significantly benefited from treatment on the scales of depression and anxiety, improvements without statistical significance were observed on the somatization and posttraumatic symptoms scales. Further studies should analyze more specifically why this patient group seemed to benefit less. Psychosomatic clinics should emphasize the role of MB in therapy, as it was shown to be an important element in the treatment process. In addition, more research is needed on the role of culturally sensitive psychotherapy, as this could lead to more acceptance and broader implementation of this concept.

## Author’s Note

The present work was performed in fulfillment of the requirements for obtaining the degree “Dr. med”.

## Data Availability Statement

The datasets generated for this study are available on request to the corresponding author.

## Ethics Statement

The studies involving human participants were reviewed and approved by Ethics Committee of the Medical Faculty of the Friedrich-Alexander University Erlangen-Nürnberg (FAU). The patients/participants provided their written informed consent to participate in this study.

## Author Contributions

FK designed the study, conducted and analyzed the data and wrote this manuscript. EM analyzed the data. YE conceived and designed the study. EM and YE provided feedback and mentorship on each stage of the research design and implementation, including a full review and provision of feedback on the final manuscript.

## Conflict of Interest

The authors declare that the research was conducted in the absence of any commercial or financial relationships that could be construed as a potential conflict of interest.
